# *In vitro* chemotherapy-associated muscle toxicity is attenuated with nutritional support, while treatment efficacy is retained

**DOI:** 10.18632/oncotarget.28279

**Published:** 2022-10-08

**Authors:** Liza A. Wijler, Francina J. Dijk, Hanil Quirindongo, Danielle A.E. Raats, Bram Dorresteijn, Matthew J.W. Furber, Anne M. May, Onno Kranenburg, Miriam van Dijk

**Affiliations:** ^1^Laboratory of Translational Oncology, Division of Imaging and Cancer, University Medical Centre Utrecht, 3584 CX, Utrecht, The Netherlands; ^2^Danone Nutricia Research, 3584 CT, Utrecht, The Netherlands; ^3^Department of Epidemiology, Julius Center for Health Sciences and Primary Care, University Medical Center Utrecht, 3584 CG, Utrecht, The Netherlands; ^4^Utrecht Platform for Organoid Technology, Utrecht University, 3584 CS, Utrecht, The Netherlands; ^*^Shared first author

**Keywords:** C2C12 myotubes, chemotherapy, chemotherapy-associated toxicity, colorectal cancer, nutrients, tumor (organoid) cells

## Abstract

Purpose: Muscle-wasting and treatment-related toxicities negatively impact prognosis of colorectal cancer (CRC) patients. Specific nutritional composition might support skeletal muscle and enhance treatment support. In this *in vitro* study we assess the effect of nutrients EPA, DHA, L-leucine and vitamin D3, as single nutrients or in combination on chemotherapy-treated C2C12-myotubes, and specific CRC-tumor cells.

Materials and Methods: Using C2C12-myotubes, the effects of chemotherapy (oxaliplatin, 5-fluorouracil, oxaliplatin+5-fluorouracil and irinotecan) on protein synthesis, cell-viability, caspase-3/7-activity and LDH-activity were assessed. Addition of EPA, DHA, L-leucine and vitamin D3 and their combination (SNCi) were studied in presence of above chemotherapies. Tumor cell-viability was assessed in oxaliplatin-treated C26 and MC38 CRC cells, and in murine and patient-derived CRC-organoids.

Results: While chemotherapy treatment of C2C12-myotubes decreased protein synthesis, cell-viability and increased caspase-3/7 and LDH-activity, SNCi showed improved protein synthesis and cell viability and lowered LDH activity. The nutrient combination SNCi showed a better overall performance compared to the single nutrients. Treatment response of tumor models was not significantly affected by addition of nutrients.

Conclusions: This *in vitro* study shows protective effect with specific nutrition composition of C2C12-myotubes against chemotherapy toxicity, which is superior to the single nutrients, while treatment response of tumor cells remained.

## INTRODUCTION

Colorectal cancer is the fifth most common type of cancer and leads to ~10% of cancer deaths [1]. Approximately 50% of late-stage colorectal cancer patients suffer from cancer-associated cachexia, which significantly contributes to cancer mortality [2, 3]. Cancer-associated cachexia is defined as ‘a multi-factorial syndrome defined by an ongoing loss of skeletal muscle mass that cannot be fully reversed by conventional nutritional support and leads to progressive functional impairment’ [4]. Cancer-associated cachexia is driven by reduced food intake and metabolic changes, including elevated energy expenditure, increased catabolism and inflammation [3, 5]. Anti-cancer treatment, like chemotherapy, can lead to additional muscle damage and accelerate muscle mass loss. This may be mediated via different mechanisms compared to cancer-related muscle loss solely, however, evidence is limited compared to publications on cancer-related muscle loss [5–8]. Chemotherapy-induced muscle loss occurs early after CRC diagnosis, and extensive muscle loss is especially observed after oxaliplatin (OX) treatment regimens [9]. Muscle loss leads to increased chemotherapy-related toxicity and is related to increased risk of dose adjustment, impaired therapy continuation and poorer prognosis [3, 10].

Several therapeutic approaches to target the multifactorial nature of cachexia are suggested in literature [3, 11–14] including adequate nutritional support, drugs to stimulate appetite and suppress inflammation, physical exercise, or a combination of all of these. A multi-model approach started at the moment of cancer diagnosis is deemed to be most effective [3, 11, 13, 15]. Adequate nutritional support can help improve lean body mass (LBM) and, therefore, could be implemented in a multimodal approach to target cachexia symptoms [5, 14, 16, 17]. These findings led to the development of a novel specialized nutritional composition (SNC), as described by Wijler et al. [18] and Van Norren et al. [17], to support the patient’s LBM and enable optimal treatment support. The SNC supports the provision of adequate caloric intake, has a L-leucine-enriched high protein content for anabolic stimulation [19], is enriched with emulsified fish oil (containing omega-3 polyunsaturated fatty acids (PUFAs) eicosapentaenoic acid (EPA) and docosahexaenoic acid (DHA)) as an anti-inflammatory agent [20–23] and vitamin D3 to support the muscle by stimulating myogenesis, cell proliferation, differentiation, regulation of protein synthesis [24]. Additionally the SNC contains oligosaccharides GOS-FOS that improve microbiota composition by enhancing immunogenic bacteria such as lactobacilli and bifidobacteria and the production of short-chain fatty acids, thereby enhancing immune function [25]. In our previous publication [18], this novel SNC showed beneficial effects on physical activity and muscle performance, without interfering the treatment response in a C26-tumor bearing mouse model supplemented with SNC. Moreover, systemic inflammation in C26-tumor bearing mice after chemotherapy treatment was reduced [18]. Furthermore, CRC-organoids were unaffected by a combination of the key nutritional ingredients (SNCi) suitable for *in vitro* analysis (i.e. EPA, DHA, L-leucine and vitamin D3), while C2C12 myotube cell viability, and protein synthesis significantly improved.

In the present study, three different *in vitro* models were used to provide better understanding and additional data on a beneficial effect of individual SNC ingredients, EPA, DHA, L-leucine and vitamin D3 or the combination (SNCi). C2C12 myotubes, C26 and MC38 colorectal cancer cells and CRC-organoids were used, in the presence or absence of chemotherapy treatment. As a marker for muscle wasting, muscle protein synthesis in C2C12 myotubes was measured. To determine chemotherapy associated toxicity, cell viability, caspase 3/7 activity (a measure of apoptosis) and LDH activity (a measure of necrotic cell death) were measured in C2C12 myotubes, and in C26 and MC38 colorectal cancer cells and CRC-organoids, cell viability was measured. While we already showed improved protein synthesis and cell viability with SNCi, we hypothesize that SNCi can counteract muscle wasting and chemotherapy-induced muscle toxicity more effectively compared to the individual SNCi ingredients EPA, DHA, L-leucine and vitamin D3 supplementation alone, providing better muscle protection, while it does not interfere with tumor organoid response to chemotherapy treatment.

## RESULTS

### Chemotherapy induces muscle toxicity in C2C12 myotubes, while anabolic capacity remains intact at low treatment doses

**Figure 1:**
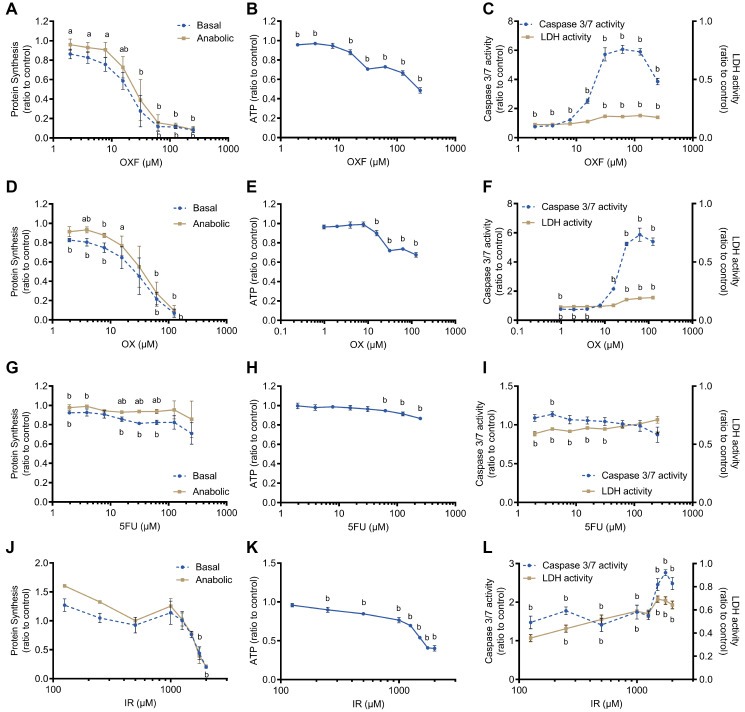
Effect of chemotherapy treatments on C2C12 myotubes. (**A**, **D**, **G**, **J**) Protein synthesis after 24 h chemotherapy treatment with oxaliplatin + 5-fluorouracil (OXF), oxaliplatin (OX), 5-fluorouracil (5FU) and irinotecan (IR). Protein synthesis was measured after chemotherapy incubation followed by 4 h deprivation of serum and L-leucine and 1 h incubation with 100 nM insulin and 5 mM L-leucine (anabolic) or without insulin and L-leucine (basal). (**B**, **E**, **H**, **K**) Cell viability after 24 h chemotherapy treatment with OXF, OX, 5FU and IR. (**C**, **F**, **I**, **L**) Caspase 3/7 activity and LDH activity after 24 h chemotherapy with OXF, OX, 5FU and IR. Values are mean ± SEM expressed as ratio to control, statistical differences were tested using a mixed model ANOVA, *post hoc* LSD (protein synthesis) or Student *t*-test (cell viability, caspase 3/7 activity and LDH activity). Significances are shown as ‘a’ = *p* < 0.05 of anabolic vs. basal condition with same chemotherapy concentration, ‘b’ = *p* < 0.05 of chemotherapy treatment vs control without chemotherapy.

Chemotherapy exposure with oxaliplatin (OX), 5-fluorouracil (5FU), a combination of oxaliplatin and 5-fluorouracil (OXF) and irinotecan (IR) of C2C12 myotubes showed a dose-dependent decline in protein synthesis (Figure 1A, 1D, 1G, 1J), ATP content (Figure 1B, 1E, 1H, 1K) and a dose-related increase in caspase 3/7 activity and LDH activity (Figure 1C, 1F, 1I, 1L) although depending on the type of chemotherapy. Chemotherapy treatment was tested in basal protein synthesis setting and upon by stimulation with insulin and L-leucine (anabolic), to investigate anabolic potential of muscle cells. Upon low dose exposure to OXF (concentrations 2.0, 3.9, 7.8 and 15.6 μM, *p* < 0.05) and OX (concentrations 3.9 and 15.6 μM, *p* < 0.05), myotubes were able to induce a significant anabolic response to insulin and leucine stimulation compared to basal protein synthesis resembling a fasting state (Figure 1A, 1D, 1J). A high dose of chemotherapy (OXF, OX, IR) significantly decreased protein synthesis and thereby the anabolic response was abolished (Figure 1A, 1D, 1J). Myotube cell viability, measured by ATP content, decreased with increasing concentrations of OXF, OX and IR, showing a characteristic sigmoid dose-response curve (Figure 1B, 1E, 1K), whereas 5FU showed significant, but limited, decline in cell viability at 250, 125 and 62.5 μM 5FU compared to vehicle control (*p* < 0.05) (Figure 1H). In line with these observations, upon chemotherapy treatment, myotubes showed significantly increased caspase 3/7 activity at higher doses of OXF, OX and IR (*p* < 0.001) (Figure 1C, 1F, 1L). Caspase 3/7 activity was unaffected by 5FU (Figure 1H). LDH release, a marker of necrotic cell death, was significant higher compared to vehicle control at higher concentrations of OXF, OX and IR (*p* < 0.005) (Figure 1C, 1F, 1L) and slightly decreased with 5FU at low dose (*p* < 0.05) (Figure 1I). Comparing the effects of chemotherapy on LDH release to caspase 3/7 activity, this data indicated that myotubes die mainly through apoptosis rather than through necrosis. In general, combination treatment of OXF showed most chemotherapy-induced toxicity in C2C12 myotubes while myotubes are the least affected by 5FU.

### Basal protein synthesis is enhanced by SNCi and single nutrients EPA, DHA, L-leucine and vitamin D3 in chemotherapy treated C2C12 myotubes

**Figure 2:**
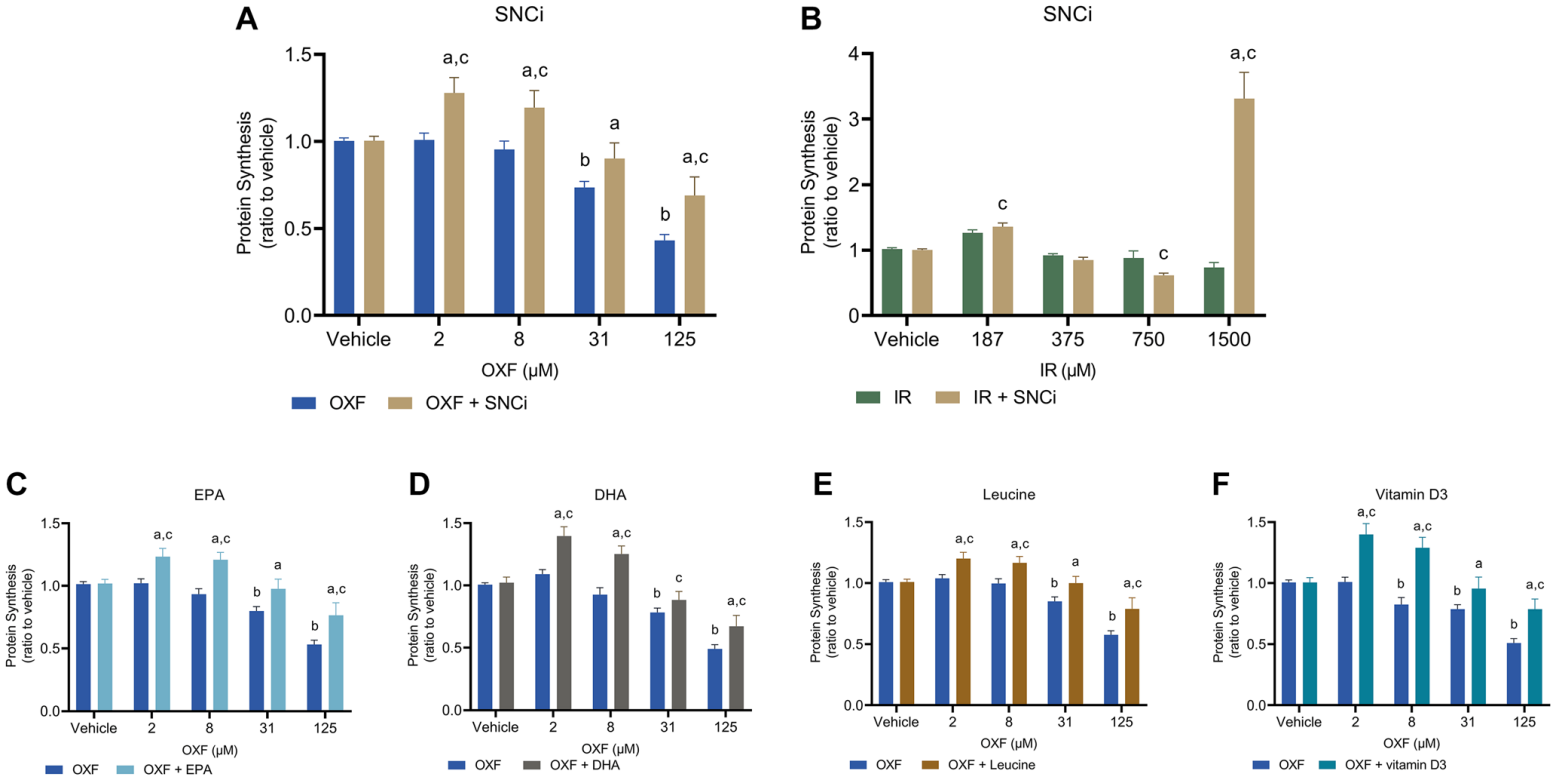
Basal protein synthesis in C2C12 myotubes after 48 h chemotherapy treatment with individual nutrients or SNCi. (**A**, **B**) C2C12 myotubes were treated with SNCi for 24 h and after this OXF or IR were added for another 24 h. Basal protein synthesis was measured after 4 h deprivation of serum and L-leucine followed by 1 h incubation without anabolic trigger of which the final 30 min in presence of 1 μM puromycin. (**C**–**F**) Effect of individual nutrients EPA, DHA, L-leucine and vitamin D3 on protein synthesis after OXF treatment. Values are mean ± SEM expressed as ratio to vehicle, statistical differences were tested using a mixed model ANOVA, *post hoc* LSD. Significances are shown as ‘a’ = *p* < 0.05 of chemo + nutrient vs. chemo control at same chemo concentration, ‘b’ = *p* < 0.05 of chemo vs. vehicle control, ‘c’= *p* < 0.05 of chemo + nutrient vs. vehicle control.

As OXF and IR treatment were most effective in inducing chemotherapy-induced toxicity in myotubes (Figure 1), the effect of SNCi was tested for its protective capacity in myotubes treated with OXF and IR. The effect of the single nutrients EPA (25 μM), DHA (12.5 μM), L-leucine (1 mM) and vitamin D3 (10 nM) were tested in myotubes treated with OXF only. A selection of four chemotherapy concentrations covering low, intermediate, and high dose, were studied. Figure 2 shows the effects of SNCi on protein synthesis at basal state in combination with OXF and IR and single nutrients in combination with OXF. Basal protein synthesis of myotubes, mimicking a fasting state, was significant increased upon SNCi incubation during OXF compared to OXF alone (*p* < 0.0001, *p* < 0.0001, *p* = 0.0043, *p* < 0.0001 respectively) (Figure 2A). Protein synthesis in IR treated myotubes was unaffected by SNCi at lower concentrations, but significantly increased at 1500 μM IR compared to the same IR concentration without SNCi (*p* < 0.0001) (Figure 2B). However, this unexpected high protein synthesis is due to the loss of many myotubes during the process of puromycin staining and should be considered as an artefact. Each individual nutrient was able to induce protein synthesis significantly in OXF treated myotubes compared to OXF treatment alone, and even with increased chemotherapy concentrations, the protective capacity of the nutrients remained intact (except with DHA at 31 μM OXF) (*p* < 0.05) (Figure 2C–2F).

### Myotube cell viability is enhanced by SNCi, EPA and vitamin D3 in chemotherapy treated C2C12 myotubes

**Figure 3:**
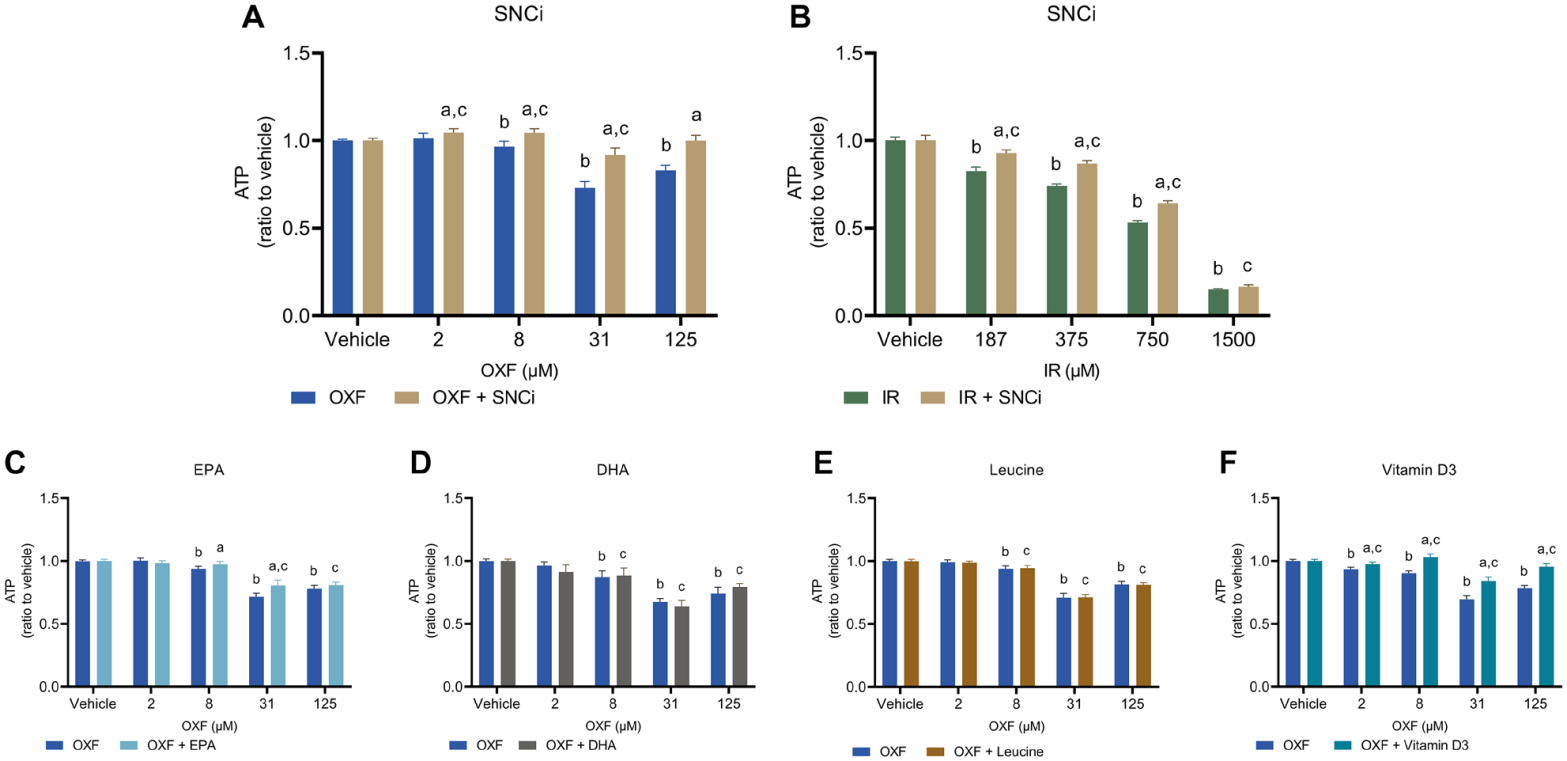
Cell viability of C2C12 myotubes after 24 h chemotherapy treatment with individual nutrients or SNCi. Cell viability was determined by measuring cellular ATP content with the CellTiter-Glo assay^®^. (**A**, **B**) Effect of SNCi on OXF and IR treated C2C12 myotubes. (**C**–**F**) Effect of individual nutrients EPA, DHA, L-leucine and vitamin D3 on protein synthesis after OXF treatment. Values are mean ± SEM expressed as ratio to vehicle, statistical differences were tested using a mixed model ANOVA, *post hoc* LSD. Significances are shown as ‘a’ = *p* < 0.05 of nutrient vs. control at same OXF concentration, ‘b’ = *p* < 0.05 of OXF vs. vehicle without nutrient, ‘c’= *p* < 0.05 of OXF vs. vehicle with nutrient.

Cell viability was assessed with the CellTiter-Glo assay measuring ATP content of the myotubes. Treatment of myotubes with SNCi and OXF showed a significant increase in cell viability at all OXF concentrations (Figure 3A) compared to OXF alone (*p* = 0.0386, *p* < 0.0001, *p* < 0.0001, *p* < 0.0001 respectively). SNCi in IR treated myotubes showed a significant increase in cell viability at all IR concentrations except 1500 μM (Figure 3B) compared to IR alone (*p* < 0.0001, *p* < 0.0001, *p* < 0.0001 respectively). Myotube cell viability significantly increased with EPA supplementation at 8 and 31 μM OXF (*p* = 0.0373, *p* < 0.0001 respectively) and vitamin D3 supplementation at 8, 31 and 125 μM OXF compared to OXF alone (all *p* < 0.0001) (Figure 3C, 3F). No effects of DHA and L-leucine alone were observed (Figure 3D, 3E) compared to OXF treatment alone.

### Effect of SNCi or single nutrient supplementation in chemotherapy treated C2C12 myotubes on caspase 3/7 activity and LDH activity

**Figure 4:**
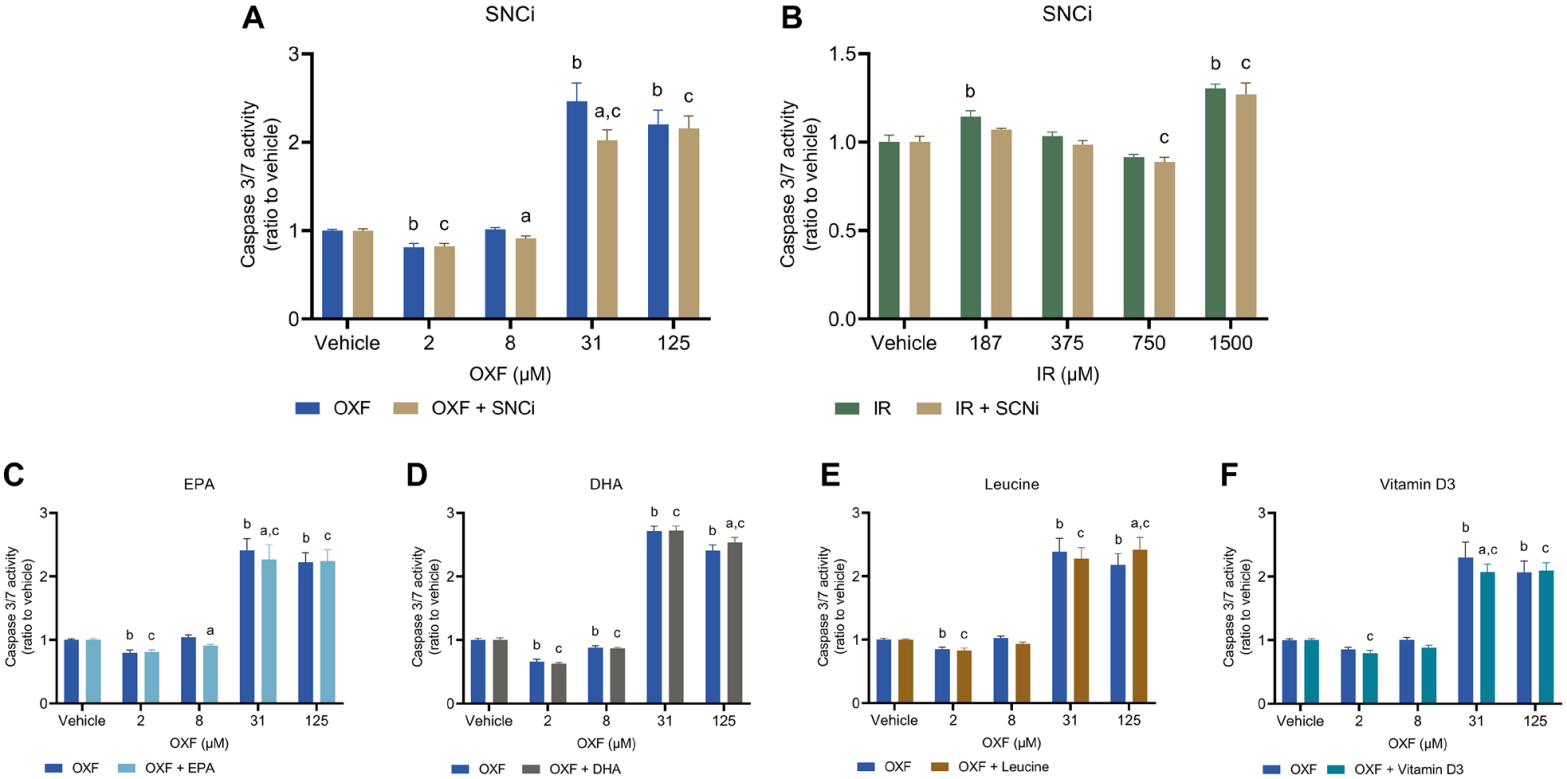
Caspase 3/7 activity of C2C12 myotubes after 24 h chemotherapy treatment with individual nutrients or SNCi. (**A**, **B**) Effect of SNCi on OXF and IR treated C2C12 myotubes. (**C**–**F**) Effect of individual nutrients EPA, DHA, L-leucine and vitamin D3 on protein synthesis after OXF treatment. Values are mean ± SEM expressed as ratio to vehicle, statistical differences were tested using a mixed model ANOVA, *post hoc* LSD. Significances are shown as ‘a’ = *p* < 0.05 of nutrient vs. control at same OXF concentration, ‘b’ = *p* < 0.05 of OXF vs. vehicle without nutrient, ‘c’= *p* < 0.05 of OXF vs. vehicle with nutrient.

**Figure 5:**
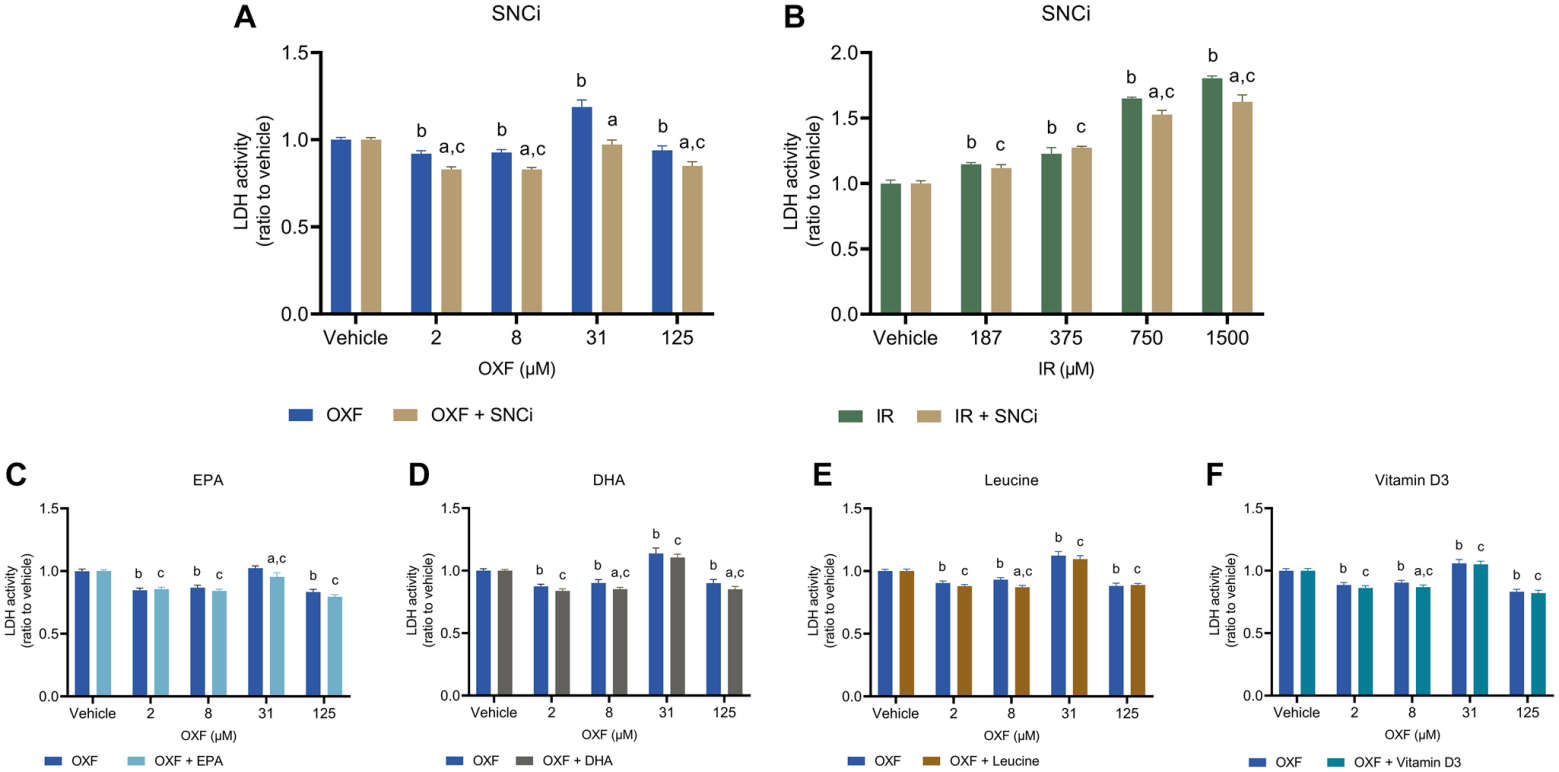
LDH activity of C2C12 myotubes after 24 h chemotherapy treatment with individual nutrients or SNCi. (**A**, **B**) Effect of SNCi on OXF and IR treated C2C12 myotubes. (**C**–**F**) Effect of individual nutrients EPA, DHA, L-leucine and vitamin D3 on protein synthesis after OXF treatment. Values are mean ± SEM expressed as ratio to vehicle, statistical differences were tested using a mixed model ANOVA, *post hoc* LSD. Significances are shown as ‘a’ = *p* < 0.05 of nutrient vs. control at same OXF concentration, ‘b’ = *p* < 0.05 of OXF vs. vehicle without nutrient, ‘c’= *p* < 0.05 of OXF vs. vehicle with nutrient.

To determine potential reduction of chemotherapy toxic effects of individual nutrients and SNCi, caspase 3/7 activity and LDH activity were assessed. Caspase 3/7 activity was used as a measure of apoptotic cell death, while LDH activity refers more to necrotic cell death. OXF with SNCi shows significant decreased caspase 3/7 activity at OXF 8 and 31 μM compared to OXF alone (*p* = 0.0454, *p* < 0.0001 respectively) (Figure 4A). IR with SNCi shows no difference between SNCi + IR and IR alone (Figure 4B). Single nutrients with OXF showed significant decrease in caspase 3/7 activity with EPA at 8 and 31 μM OXF (*p* = 0.0137, *p* = 0.0072 respectively) and with vitamin D3 at 31 μM OXF (*p* = 0.0041) compared to OXF alone (Figure 4C, 1F). An increase of caspase 3/7 activity was observed with DHA and L-leucine at 125 μM OXF compared to OXF alone (Figure 4D, 4E) (*p* = 0.0134, *p* = 0.0011 respectively). LDH activity significantly decreased with SNCi in OXF treated myotubes compared to OXF alone at all four concentrations (*p* < 0.0001) (Figure 5A), however in IR treated myotubes, SNCi decreased LDH activity at 750 and 1500 μM IR) (*p* = 0.0023, *p* < 0.0001 respectively) (Figure 5B). Single nutrients were less effective in decreasing LDH activity, EPA at 31 μM OXF (*p* = 0.0007), DHA at 8 and 125 μM OXF (*p* = 0.0257, *p* = 0.0238 respectively) L-leucine and vitamin D3 at 8 μM OXF showed a significant decrease in LDH activity (Figure 5C–5F) (*p* = 0.0266) compared to control.

### Single nutrient supplementation or combination does not interfere with chemotherapy responses in conventional 2D and innovative 3D *in vitro* models for colorectal cancer

To investigate the potential effects of individual nutrients and SNCi in combination with chemotherapy on tumor cell viability, a panel of *in vitro* tumor models was used. The murine C26 and MC38 adenocarcinoma cell lines are classical examples of commonly used *in vitro* colorectal cancer models, and the C26 cell line is a widely used cell line for tumor induction in cachectic mouse models [26]. Recently, innovative organoid technology enabled the use of patient-derived 3D cultures to assess individual patient responses to chemotherapy *in vitro* and capture patient heterogeneity [27]. In this study, a panel of five patient-derived CRC-organoids (PDO1 – PDO5) and four murine-derived CRC-organoids (MDO1 – MDO4) were used, creating a diverse *in vitro* drug screening tumor organoid panel.

**Figure 6:**
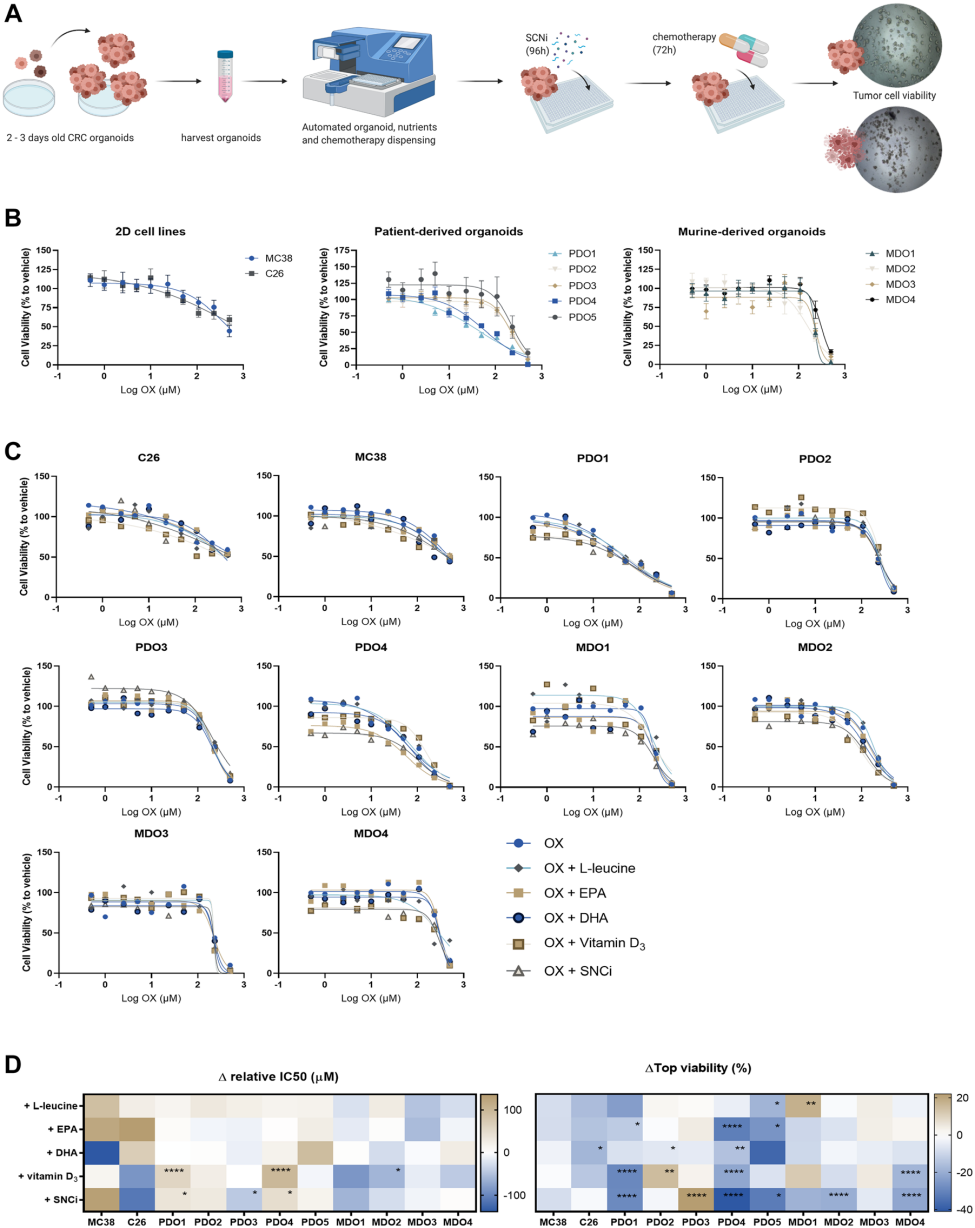
Cell viability of colorectal cancer (CRC) *in vitro* tumor models after chemotherapy treatment with individual nutrients and SNCi. (**A**) Experimental overview, expansion of organoids after 2–3 days after single-cell making, harvesting and dispensing organoids in 384 wells plates. Recovery overnight, followed by automatic dispensing of SNCi nutrients (96 h incubation) and oxaliplatin chemotherapy (72 h incubation). Cell viability measured by Cell Titer Glo 2.0 assay. (**B**) Cell viability curves of OX treated cell lines, PDOs and MDOs. Cell viability (%) is expressed as relative to vehicle without chemotherapy treatment or nutritional support. (**C**) Individual cell viability curves of colorectal cancer (CRC) *in vitro* tumor models treated with OX and individual nutrients or SNCi. Cell viability (%) is expressed as relative to vehicle without chemotherapy treatment or nutritional supplementation. (**D**) Delta values of relative IC_50_ (μM) and top viability (%) of *in vitro* tumor models after supplementation with L-leucine, EPA, DHA, vitamin D3 or SNCi. IC_50_ values are expressed as relative IC_50_ values, as not all curves start at 100% viability. Top viability represents the highest cell viability value in the curve (usually at low concentrations) and values with nutritional supplementation are expressed as delta to OX treatment alone. Abbreviations: PDO: patient-derived CRC tumor organoids; MDO: mouse-derived CRC tumor organoids. ^*^ *p* < 0.05; ^**^ *p* < 0.01; ^***^ *p* < 0.001; ^****^ *p* < 0.0001.

In Figure 6A, the experimental workflow of *in vitro* drug screening is presented. All *in vitro* tumor models were treated with a concentration series of OX and cell viability curves were plotted, revealing heterogeneous chemotherapy responses among the different *in vitro* tumor models (Figure 6B). Combination of 5FU with OX treatment did not lead to enhanced killing response compared to single OX treatment (data not shown), therefore *in vitro* tumor models were treated with OX only to reach maximal inhibition of cell viability with highest dose 500 μM OX instead of 250 μM OX + 250 μM 5FU, as technical limitations prevented treating with higher chemotherapy concentrations. To assess the effects of individual nutrients and SNCi in combination with chemotherapy, tumor cell viability curves of OX with and without nutritional support were plotted (Figure 6C). IC_50_ and top viability values were extrapolated (Supplementary Table 1). Although relative IC_50_ values of PDO1 and PDO4 were significantly increased upon supplementation with vitamin D_3_ and SNCi, relative IC_50_ values of 9 out of 11 models were not significantly affected by nutritional supplementation (Figure 6D). The relative IC_50_ value of PDO3 and MDO3 even significantly decreased upon vitamin D3 or SNCi supplementation compared to OX treatment alone. When investigating top viability values of killing curves, 95% of tested nutritional interventions resulted in non-affected or significantly decreased top viability, whereas SNCi showed most pronounced effects compared to individual ingredients (Figure 6D).

**Figure 7:**
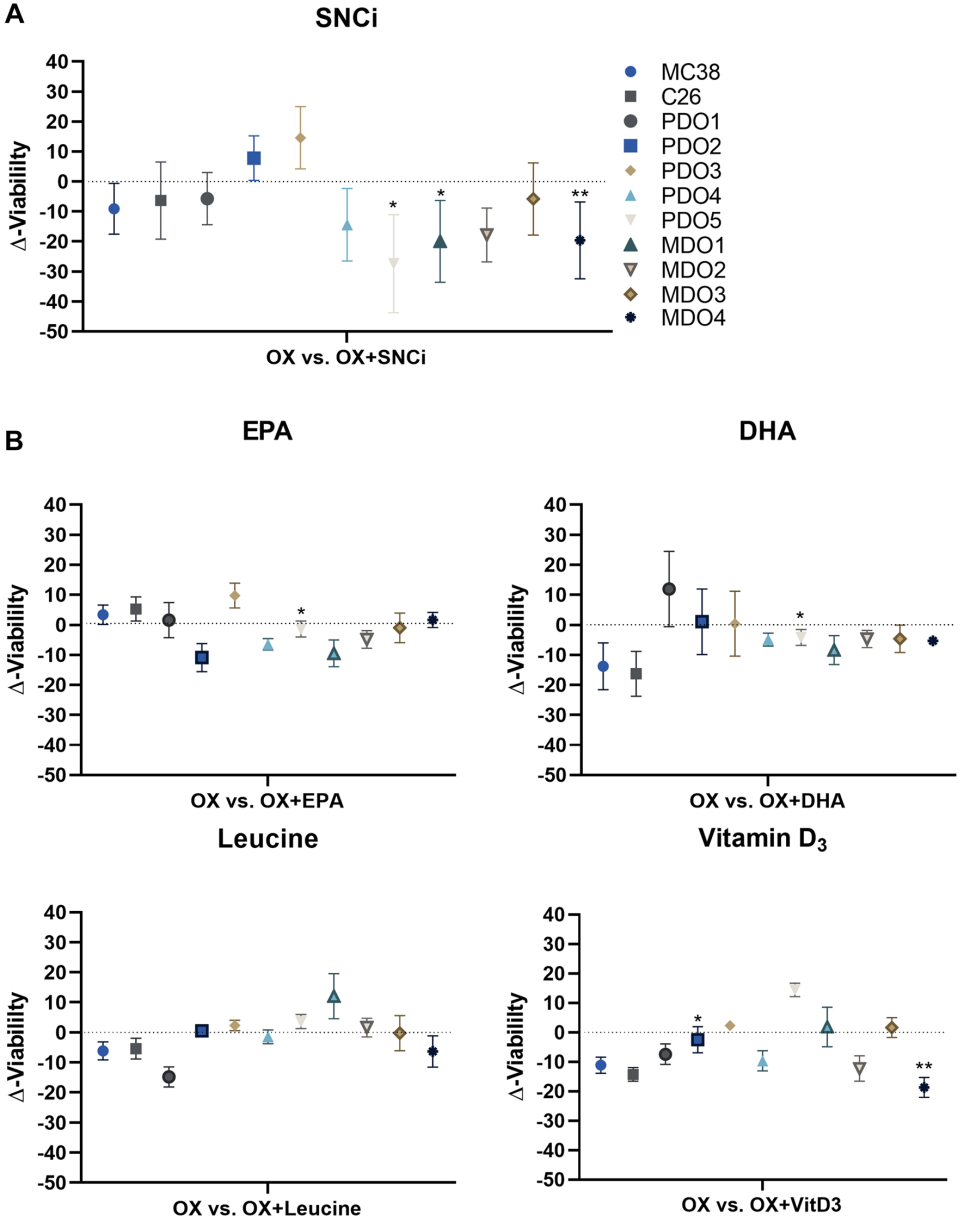
Delta cell viability of colorectal cancer (CRC) *in vitro* tumor models after chemotherapy treatment with individual nutrients and SNCi. (**A**) Averages of delta cell viability (Δ-viability) of individual *in vitro* tumor models after supplementation with SNCi. (**B**) Averages of delta cell viability (Δ-viability) of individual *in vitro* tumor models after supplementation with EPA, DHA, L-leucine and vitamin D3. Significances are represented as ^*^ *p* < 0.05; ^**^ *p* < 0.01; ^***^ *p* < 0.001; ^****^ *p* < 0.0001. Abbreviations: PDO: patient-derived CRC tumor organoid; MDO: murine-derived CRC tumor organoid.

After investigating the IC_50_ values and top viability, we assessed the effects of nutritional supplementation on the total cell viability curve by expressing the delta viability compared to OX treatment alone. Averaged tumor cell viability remained unchanged for MC38, C26, PDO4, MDO2 and MDO3 (Figure 7A). In three models, PDO5, MDO1 and MDO4, averaged tumor cell viability even significantly decreased upon SNCi supplementation during OX treatment (*p* = 0.014, *p* = 0.015 and *p* = 0.007). PDO2 and PDO3 showed non-significantly increased cell viability (*p* = 0.075 and *p* = 0.123). The generalized effect of SNCi on all *in vitro* tumor models combined showed a Δ-viability of −9.4% with a standard deviation of 11.2%. When combining OX treatment with single nutrient supplementation of EPA, DHA, L-leucine and vitamin D3, Δ-viability values remained unaffected or even decreased (Figure 7B), although differences in responses between *in vitro* tumor models were observed. The generalized effect of single nutrients on *in vitro* tumor models showed Δ-viability of −1.2% ± 11.9% for EPA, −4.5% ± 19.5% for DHA, −1.3% ± 11.3% for L-leucine and −5.1% ± 10.9% for vitamin D3, indicating nutrient supplementation does not significantly influence treatment efficacy of chemotherapy treatment on the *in vitro* tumor panel.

To provide insights in the potential effects of SNCi on downstream proliferation and metabolic pathways of *in vitro* tumor models, bulk RNA sequencing was performed on PDOs treated with 5 μM OX alone or in combination with SNCi. Molecular gene set analyses of proliferative and metabolic pathways revealed that no significant differences were found upon supplementation with SNCi in RNA expression (data not shown).

## DISCUSSION

In this *in vitro* study, supplementation of single nutrients EPA, DHA, L-leucine and vitamin D3 attenuated chemotherapy-associated toxicity of C2C12 myotubes as shown by an enhanced basal protein synthesis, increased cell viability with EPA and vitamin D3, with varying effects on caspase 3/7 activity and LDH activity. These effects were further strengthened upon supplementation with SNCi, indicating that the combination of supporting nutrients is essential to deliver maximum benefit to chemotherapy treated C2C12 myotubes. Neither supplementation with single nutrients nor SNCi interfered with chemotherapy treatment efficacy in a large series of CRC tumor cells and organoids. Moreover, SNCi did not alter RNA expression of molecular gene set pathways in CRC patient-derived organoids. This study focusses on the *in vitro* effects of nutrients and the nutritional combination SNCi in muscle and tumor cells, not capturing the complexity of the whole-organ or whole-organism. Though, this *in vitro* study was followed up by a cachexia mouse-model where we tested the same nutritional combination [18].

The effects of four chemotherapeutic regimens used as standard of care treatment for CRC were tested on C2C12 myotubes and *in vitro* CRC tumor models. While treatment with OX, OXF and IR showed a significant decline in protein synthesis, myotube cell viability and increased caspase 3/7 activity and LDH activity, the effects of 5FU were modest. In line with clinical practise, 5FU is often combined with OX or IR in FOLFOX or FOLFIRI treatment regimens and shows limited efficacy as monotherapy in CRC patients with advanced disease [28]. Experiments with C2C12 myotubes with chemotherapy and nutrients were therefore performed with the combination of oxaliplatin and 5-fluorouracil (OXF) and SNCi with irinotecan (IR). Experiments with C26 and MC38 colorectal cancer cells and CRC-organoids were performed with oxaliplatin alone since the addition of 5FU did not enhance the killing response.

Basal protein synthesis in C2C12 myotubes was increased with all single nutrients, as well as SNCi, implying that these nutrients are capable of inducing protein synthesis without addition of the anabolic trigger insulin and leucine. Cell viability was increased with EPA and vitamin D3 and the combination SNCi, but not with leucine and DHA. In the same line of expectation, LDH activity was decreased with SNCi but not with all single nutrients, this was less pronounced in caspase 3/7 activity. These results suggest superior results with SNCi over the single nutrients, improved protein synthesis and cell viability with less cell death in OXF treated myotubes.

The results are in line with our previous publication [18], where beneficial effects were described on muscle function and physical activity in the C26 tumor-bearing mouse model supplemented with SNC (SNCi with additional high protein content and GOS-FOS) treated with chemotherapy. In the current *in vitro* model, no systemic effects could be determined but provides additional support specific on muscle function (i.e., protein synthesis) and chemotherapy-related toxicity (i.e., viability and toxicity) of both muscle cells and tumor cells.

OXF treatment caused most chemotherapy-induced toxicity in myotubes, reflective of colorectal patient responses observed in the clinic [10]. Chemotherapy treatment decreased protein synthesis, cell viability and increased apoptosis and necrosis in myotubes at higher concentrations, though, at low chemotherapy concentrations myotubes were still capable of producing anabolic responses, creating opportunity for testing nutritional supplementation of EPA, DHA, L-leucine and vitamin D3 and the combination of these nutrients, SNCi. OXF targets actively proliferating cells by induction of apoptosis and DNA damage [29]. Since *in vitro* C2C12 myotubes are differentiated and therefore do not proliferate, the detrimental effects of chemotherapy are expected to be smaller compared to regenerating muscle tissues *in vivo,* where muscle satellite cells act as stem cells renewing muscle tissue [30]. Although chemotherapy concentrations that effectively resulted in C2C12 myotube toxicity were relatively high compared to generally observed plasma levels in cancer patients undergoing chemotherapy treatment [31, 32], muscle loss as a result of chemotherapy is still widely observed in CRC patients [10]. Therefore, we argue anabolic responses of skeletal muscles of the patients are targetable for improving via nutritional interventions like SNCi.

Our *in vitro* results complement evidence found in clinical studies investigating the effects of nutritional support on cachectic cancer patients. For instance, Deutz *et al.* found that muscle protein synthesis was enhanced with protein-enriched specialized nutrition in cachectic cancer patients [16]. In a small clinical study, Bauer *et al.* [33] demonstrated improved LBM, physical performance status and quality of life in cachectic pancreatic and non-small-cell lung cancer patients receiving chemotherapy who were supported with an energy dense, protein and EPA-enriched supplement [33–35]. This was also confirmed in two larger randomized clinical trials (RCT) with non-small-cell lung cancer patients [34, 35]. De van der Schueren et al. [36] showed in a systematic review of RCTs an overall positive effect of nutritional interventions during chemo(radio)therapy on body weight, while also expressing the need for additional RCTs investigating nutritional interventions on clinical outcomes with the most promising nutrients.

General nutritional support may not be sufficient to benefit all cachectic cancer patients due to their metabolic malfunctioning and cachexia stage. This provides a need for specialized nutritional interventions to support to this specific patient population. While increasing evidence shows that general nutritional support may not benefit all cachectic patients due to metabolic malfunctioning, specialized nutritional interventions seem promising. Our results confirm that single nutrient interventions, such as only protein, PUFA or vitamin D3 supplementation are not as effective in attenuating cancer cachexia as combination multiple active nutrients [13, 14, 17, 19, 36–38]. Whereas our *in vitro* data do provide useful insights on the direct effects of SNCi on myotube performance, there are limitations of *in vitro* models towards clinical translation. While we have used state-of-the-art 3D tumor models, *in vitro* muscle assays still rely for the majority on 2D cell lines. Moreover, *in vitro* assays do not capture the complexity of whole-organ or whole-organism, including tumor or muscle micro environmental cues, gastrointestinal modifications of nutritional ingredients and immune system interactions. Therefore, confirmation of our *in vitro* outcomes and further investigation of SNCi with *in vivo* testing and RCTs with chemotherapy-treated cachectic colorectal cancer patients remains indispensable [39, 37]. Moreover, multi-modal therapeutic approaches could enhance clinical outcomes even more than single interventions. By combining dietary interventions with exercise and/or pharmaceutical interventions with orexigenic agents, such as ghrelin and anamorelin [5, 14, 16], cancer cachexia could be targeted.

To summarize, the combination of nutrients EPA, DHA, L-leucine and vitamin D3 (SNCi) reduced muscle wasting as shown by enhanced (basal) protein synthesis and attenuated chemotherapy associated toxicity of myotubes as shown by an increased cell viability, decreased cell death (LDH activity). Importantly, the nutrient combination did not interfere with chemotherapy treatment efficacy in tumor cells and organoids. Our *in vitro* study demonstrates the importance of specialized nutritional support and combining multiple nutrients to reach maximum effect to attenuate chemotherapy-associated muscle toxicity of skeletal muscle in cancer patients.

## MATERIALS AND METHODS

### C2C12 cell culture

C2C12 mouse myoblasts (provided by Maastricht University) were cultured in culture medium (DMEM with high glucose (Sigma) and supplemented with 2.4 g/L NaHCO_3_, 10% Foetal Bovine Serum and 1% Penicillin-Streptomycin). Cells were cultured twice a week until maximal confluency of about 70%. For differentiation into myotubes, cells were plated in 96-well plates at 1.0e^5^ cell/mL (100 μL per well). The next day, culture medium was replaced by differentiation medium (DMEM-high glucose, supplemented with 2.4 g/L NaHCO_3_, 2% Horse Serum (heat inactivated) and 1% Penicillin-Streptomycin). Within 5 days, myoblasts were differentiated into myotubes and exposed to chemotherapy for 24 h, or pre-incubated with nutritional ingredients for 1 h or 24 h prior to 24 h chemotherapy treatment.

### Tumor organoid culture

Patient-derived CRC-organoids were previously established and characterized [40]. CRC organoids were cultured in droplets of Growth Factor Reduced Basement Membrane Extract (BME; Amsbio). Human CRC organoid culture medium contained Advanced Dulbecco’s modified Eagle medium (DMEM)/F12 (Invitrogen) with 1% Penicillin-Streptomycin (Gibco), 1% HEPES buffer (Invitrogen) and 1% Glutamax (Invitrogen), 10% Noggin conditioned medium (produced by lentiviral transfection), 2% B27 supplement (Invitrogen), 1.25 mM n-Acetylcysteine (Sigma-Aldrich), 10 mM Nicotinamide (Sigma-Aldrich), 500 nM A83-01 (Tocris) and 10 mM SB202190 (ApexBio).

Murine tumor organoids were derived from spontaneously formed colorectal tumors in a transgenic mouse model with conditional activation of the Notch1 receptor and deletion of p53 in the digestive tract [41]. Exome sequencing revealed that all tumors harbor mutations in either the *Ctnnb1* or *Apc* genes, demonstrating classical Wnt pathway activation [41]. CRC organoids were cultured in Advanced DMEM/F12 (Thermo Fisher Scientific), supplemented with 1% Penicillin-Streptomycin (Gibco), 1% HEPES buffer (Invitrogen), 2 mM Glutamax (Invitrogen), 2% B27 supplement (Invitrogen), 100 ng/ml Noggin (produced by lentiviral transfection), 10 nM murine recombinant FGF (PeproTech) and 1 mM n-Acetylcysteine (Sigma-Aldrich). Organoids were passaged once per week through TrypLE Express (Invitrogen) treatment combined with mechanic disruption.

### Murine colon adenocarcinoma cell lines C26 and MC38

Murine colon adenocarcinoma cell lines C26 and MC38 were cultured in adherent 10 cm dishes (Corning) and maintained in DMEM high glucose (Invitrogen), supplemented with 1% penicillin–streptomycin, 1% Glutamax and 10% heat-inactivated Foetal Bovine Serum (FBS) (Invitrogen). Cell lines were passaged twice a week. Medium was refreshed twice a week or at indication. All cultures were maintained at 37°C in a humidified atmosphere containing 5% CO_2_ and have repeatedly been tested negative for mycoplasma.

### Key nutritional ingredients

Four SNC key nutritional ingredients that allowed *in vitro* testing were tested as single nutrients or as a mix of all four nutrients (SNCi). These nutrients were tested in a final concentration of 25 μM eicosapentaenoic acid (EPA) (Sigma-Aldrich), 12.5 μM docosahexaenoic acid (DHA) (Sigma-Aldrich), 1 mM L-leucine (Sigma-Aldrich) and 10 nM Vitamin D3 (Sigma-Aldrich or Cayman). Chosen concentrations of EPA and DHA were based on previous data showing significant increase in protein synthesis in C2C12 myotubes. L-Leucine concentration of 1 mM was based on previous data showing increased protein synthesis in C2C12 myotubes. Vitamin D3 concentration of 10 nM was selected because of Salles et al. [42] showed significant increase in protein synthesis with 1 and 10 nM vitamin D3 together with insulin and leucine as anabolic stimuli. For muscle cell assays, EPA and DHA were dissolved in 100% ethanol and further diluted in PBS + 2.5% essentially fatty acid free BSA (Sigma-Aldrich), vitamin D3 was dissolved in 100% ethanol, L-leucine in differentiation medium. For tumor cell and organoid screening L-leucine was dissolved in PBS-Tween (0.1%); EPA, DHA and Vitamin D3 were dissolved in DMSO.

### Chemotherapeutic agents

Chemotherapy exposure was performed in a dose-response curve with 5-fluorouracil (5FU, Sigma)), oxaliplatin (OX, Selleck Chemicals), a 1:1 combination of 5FU and OX (OXF) or irinotecan (IR, Selleck Chemicals). Stocks were made in DMSO and stored at −20°C until use.

### ATP content, caspase 3/7 activity and LDH activity of C2C12 myotubes

C2C12 myotubes were cultured in white 96-well plates with clear bottom as described above. After 1 h pre-treatment with nutrients and 24 h with nutrients and chemotherapy, ATP content was assessed by the CellTiter-Glo Assay^®^ (Promega) as an indicator of cell viability. Briefly, myotubes were incubated for 30 min at RT before 100 μl CellTiter-Glo substrate was added to each well. Cells were incubated for 10 min at RT and luminescence was measured using a FlexStation^®^ Microplate Reader (Molecular Devices). A separate well plate was used to assess caspase 3/7 activity and LDH activity. First, 50 μL medium of each well was transferred into a new transparent plate for the measurement of LDH release. Caspase-Glo 3/7 Assay^®^ (Promega) was used to measure caspase 3/7 activity by adding 50 μL of substrate to each well. Cells were incubated for 30 min at RT and luminescence was measured using the FlexStation^®^ Microplate Reader. In the transparent plate with 50 μL medium LDH activity was assessed using the Cytotoxicity Detection Kit (LDH, Roche) by adding 50 μL of reagent to each well and incubate for 30 min at RT in a dark surrounding. Thereafter absorbance was measured at 490 nm and background correction at >600 nm using the FlexStation. Values of all assays were expressed as a ratio to vehicle treated cells. All experiments were repeated at least three times, with a minimum of 5 replicates per experiment.

### Protein synthesis of C2C12 myotubes

C2C12 myotubes were cultured in black 96-well plates with clear bottom as described above. Protein synthesis of myotubes was measured using the SUnSET method adapted from Goodman et al. [43]. After 24 h incubation with nutrients followed by 24 h incubation with nutrients and chemotherapy, the myotubes were placed on L-leucine-and serum-free medium for 4 h. Subsequently, cells were incubated with 100 nM insulin (Sigma) and 5 mM L-leucine for anabolic stimulation, or without insulin and L-leucine for basal protein synthesis. After 30 min, 1 μM puromycin (Calbiochem) was added for 30 min to label newly synthesized proteins, followed by fixation with 4% PFA (Merck) for 20 min. Then cells were washed 3 times with PBS to remove excess PFA and permeabilized with 0.5% Triton X-100 (Sigma-Aldrich) for 5 min. After washing again, the cells were blocked with block buffer (PBS (Invitrogen) with 3% goat serum (Chemicon International) and 0.1% Triton X-100) for 1 h. Cells were incubated overnight with anti-puromycin antibody (clone 12D10, 1:200, Millipore) in PBS with 3% goat serum and secondary antibody (Anti-mouse IgG2a DyLight 488, 1:200, Molecular Probes) in PBS with 0.05% BSA (Sigma-Aldrich) for 1 h. After washing, 50 μL mounting medium AF1 (CitiFluor) per well was added and fluorescence was measured with excitation at 478 nm and emission at 518 nm with the FlexStation. After fluorescent measurement, protein content was determined with an amido black assay [44]. Plates were incubated with amido black reagent (0.1% amido black (Sigma-Aldrich) dissolved in sodium acetate buffer (Sigma-Aldrich) and filtered) for 30 min and with acidic water to remove excess amido black staining. 150 μl NaOH was added to elute protein bound amido black, incubated for about 15 min to dissolve the amido black while mixing the plate. Absorbance was measured at 620 nm with background corrected at 405 nm. Protein synthesis rate was obtained by dividing RLU values of puromycin signal by the corresponding absorption values of the amido black signal. To compare individual experiments, a ratio was calculated between each condition and vehicle treated cells, where the vehicle was set to 1. All experiments were repeated at least three times, with a minimum of 5 replicates per experiment.

### Cell viability of tumor cell lines and tumor organoids

C26 cells, MC38 cells and tumor organoids were cultured as described above. For tumor cell lines, 1000 single cells were suspended in 40 μL culture medium and dispensed in a 384-well plate (CLS3571; Corning) using the Multidrop™ Combi Reagent Dispenser with a small tubing cassette (ThermoFisher Scientific). For tumor organoids, a bottom layer of 10 μL BME was dispensed in a 384-well plate using the Multidrop™, centrifuged and kept at RT for 15-25 min to allow full polymerisation of BME. Depending on proliferation rate, approximately 750-1000 3-day-old CRC-organoids suspended in 40 μL basal culture medium were dispensed on top of BME and briefly centrifuged (1000 rpm at 4°C).

Both organoids and cell lines settled for 4 h before exposure to SNCi, and 24 h later exposed to chemotherapeutic OX. Both SNCi and chemotherapeutic were added using HP D300e digital dispenser (TECAN), non-treated wells received normalisation with DMSO. After exposure to SNCi (96 h) and chemotherapy (72 h), tumor organoid viability was quantified using CellTiter-Glo 2.0 Luminescence Assay^®^ (Promega) and SpectraMax M5e reader (Molecular Devices). For *in vitro* tumor model, experiments were executed at least three times, with a minimum of three replicates per experiment. A four-parameter non-linear mixed-effect model was used to fit dose-response curves in the presence or absence of individual nutrients or SNCi. The formula of the four-parameter non-linear mixed-effect models was as follows: Cell viability % = ((top − bottom)/(1 + exp(−(log[IC_50_] − log([chemotherapy]))/slope))) + bottom. The fixed-effect bottom parameter estimates were constrained at 0% (i.e., bottom was fixed at zero. Top viability, IC_50_ values and 95% confidence intervals were calculated. Delta viability was expressed as average cell viability relative to OX treatment alone.

### RNA sequencing

Tumor organoids were expanded for three days, plated in BME droplets of 10μL, pre-treated with SNCi (24 h) and treated with 5 μM oxaliplatin (72 h). After 96 h, organoids were harvested using dispase (ThermoFisher Scientific) and spun down (1 min, 14000 × g) in a pellet, and snap frozen in liquid nitrogen. RNA was isolated using RNeasy Mini Kit (Qiagen) and RNA quality was checked using 2100 BioAnalyzer (Agilent). Briefly, sequencing libraries were made from poly-adenylated RNA using the Rapid Directional RNA-Seq Kit (NEXTflex) and sequenced on Illumina NextSeq500 to produce single-end 75 base long reads (Utrecht Sequencing Facility). Reads were aligned to human reference genome GRCh37 and pathway analysis (Geneset Maps) was performed using R2 software [45].

### Statistical analysis

All data are expressed as mean ± SEM. Statistical analysis was performed using IBM SPSS Statistics (version 19; SPSS Inc. Chicago, IL, USA). A Student’s *t*-test or mixed model ANOVA followed by *post hoc* LSD analysis were used to compare different treatments. Statistical difference was reached with *P* < 0.05. Figures were created using BioRender (https://biorender.com), Illustrator (Version 25.2.1) and GraphPad (Version 8.3.4).

## SUPPLEMENTARY MATERIALS



## Abbreviations

ATP: adenosine triphosphate
BME: basement membrane extract
CRC: colorectal cancer
DHA: docosahexaenoic acid
DMSO: dimethyl sulfoxide
EPA: eicosapentaenoic acid
5FU: 5-fluorouracil
FOLFOX: 5-fluorouracil, oxaliplatin, leucovorin
FOLFIRI: 5-fluorouracil, irinotecan, leucovorin
GOS-FOS: galacto-oligosaccharides and fructo-oligosaccharides
IC_50_: half-maximal inhibitory concentration
IR: irinotecan
LBM: lean body mass
LDH: lactic acid dehydrogenase
MDO: mouse derived organoid
PUFAs: polyunsaturated fatty acids
SNC: specialized nutritional composition
SNCi: specialized nutritional composition ingredients (combination)
SUnSET: surface sensing of translation
OX: oxaliplatin
OXF: oxaliplatin and 5-fluorouracil
PDO: patient derived organoid
