# Supplementary material for: In vitro chemotherapy-associated muscle toxicity is attenuated with nutritional support, while treatment efficacy is retained
Source: Oncotarget. 2022 Oct 8;13:1094–108. doi: 10.18632/oncotarget.28279 (PMC9564364; doi:10.18632/oncotarget.28279)
Supplement: Supplementary file 1 [file oncotarget-13-28279-s001.pdf]

In vitro chemotherapy-associated muscle toxicity is attenuated with nutritional support, while treatment efficacy is retained

SUPPLEMENTARY MATERIALS

Supplementary Table 1: IC_{50} values and top viability (%) of cell viability curves of CRC *in vitro* tumor models when treated with OX, in combination with L-leucine, EPA, DHA, vitamin D3 or SNCi

Δ IC_{50} (µM)	MC38	C26	PDO1	PDO2	PDO3	PDO4	PDO5	MDO1	MDO2	MDO3	MDO4
OX	405	364,9	42,29	232,5	214,3	56,56	221,8	225,1	115,2	235,4	306,1
OX + L-leucine	88,1	23,8	14,21	30,7	22,1	15,48	17,4	–25,5	27,7	–50,1	–28,9
OX + EPA	121,5	135,9	4,93	–8,8	–11,3	1,56	24,7	–17,5	3,8	–57,1	–9,4
OX + DHA	–138,9	68,7	–0,69	2,1	–13,7	17,4	91,3	2,5	–29,8	–4,2	25,1
OX + $VitD_3$	13,8	–88,9	57,28	23,6	–0,1	97,34	–8,5	–87,6	–62,09	–16,5	–44,1
OX + SNCi	131,5	–112,6	36,99	30,5	–45,3	45,94	16,4	–62,8	–29	–9,9	3,1

Δ IC_{50} (µM)	MC38	C26	PDO1	PDO2	PDO3	PDO4	PDO5	MDO1	MDO2	MDO3	MDO4
OX + L-leucine	n.s.	n.s.	n.s.	n.s.	n.s.	n.s.	n.s.	n.s.	n.s.	n.s.	n.s.
OX + EPA	n.s.	n.s.	n.s.	n.s.	n.s.	n.s.	n.s.	n.s.	n.s.	n.s.	n.s.
OX + DHA	n.s.	n.s.	n.s.	n.s.	n.s.	n.s.	n.s.	n.s.	n.s.	n.c.	n.s.
OX + Vit D_3	n.s.	n.s.	****	n.s.	n.s.	****	n.s.	n.s.	*	n.c.	n.s.
OX + SNCi	n.s.	n.s.	*	n.s.	*	*	n.s.	n.s.	n.s.	n.c.	n.s.

Δtop Viability (%)	MC38	C26	PDO1	PDO2	PDO3	PDO4	PDO5	MDO1	MDO2	MDO3	MDO4
OX	107,6	120,2	106,2	97,38	102,7	107,9	122,6	96,51	101,3	88,3	101,2
OX + L-leucine	–8,8	–16,5	–24,13	2	1	–4,3	–18,6	17,59	–1,39	3,93	–3,43
OX + EPA	–8,61	–15	–12,98	–1,04	4,3	–31,67	–26,44	–10,16	–7,08	3,7	2,1
OX + DHA	–3,3	–18,2	–7,62	–6	–5,21	–15,32	–37,5	–9,83	–2,66	–4,6	–7,11
OX + Vit D_3	–1,1	–13,8	–29,44	14,92	2,7	–21,88	–7,2	11,69	–8,37	4,79	–18,38
OX + SNCi	–8,45	–1,6	–29	–2,32	22	–41,08	–32,33	–19,04	–20,14	–5,65	–21,98

Δtop Viability (%)	MC38	C26	PDO1	PDO2	PDO3	PDO4	PDO5	MDO1	MDO2	MDO3	MDO4
OX + L-leucine	n.s.	n.s.	n.s.	n.s.	n.s.	n.s.	*	**	n.s.	n.s.	n.s.
OX + EPA	n.s.	n.s.	*	n.s.	n.s.	****	*	n.c.	n.s.	n.s.	n.s.
OX + DHA	n.s.	*	n.s.	*	n.s.	**	n.s.	n.c.	n.s.	n.c.	n.s.
OX + Vit D_3	n.s.	n.s.	****	**	n.s.	****	n.s.	n.s.	n.s.	n.c.	****
OX + SNCi	n.s.	n.s.	****	n.s.	****	****	*	n.c.	****	n.c.	****

Top viability represents the highest cell viability value in the curve (usually at low concentrations) and values with nutritional supplementation are expressed as delta to OX treatment alone. IC50 values are expressed as relative IC50 values, as not all curves start at 100% viability. Cell viability (%) is expressed as relative to vehicle without chemotherapy treatment or nutritional support. $^{*}p < 0.05$; $^{**}p < 0.01$; $^{***}p < 0.001$; $^{****}p < 0.0001$; Abbreviations: n.s.: not significant; n.c.: not computed as confidence intervals could not sufficiently be calculated from curve fitting.
